# The Role of Morbid Obesity in the Promotion of Metabolic Disruptions and Non-Alcoholic Steatohepatitis by Helicobacter Pylori

**DOI:** 10.1371/journal.pone.0166741

**Published:** 2016-11-28

**Authors:** Albert Lecube, Silvia Valladares, Carolina López-Cano, Liliana Gutiérrez, Andreea Ciudin, José Manuel Fort, Josep Maria Reñé, Xavier Matias-Guiu, Inés de Torres, Marta Bueno, Judit Pallarés, Juan Antonio Baena

**Affiliations:** 1 Endocrinology and Nutrition Department, EASO Collaborating Centre for Obesity Management, Arnau de Vilanova University Hospital, Institut de Recerca Biomèdica (IRB) and University of Lleida, Lleida, Spain; 2 CIBER de Diabetes y Enfermedades Metabólicas asociadas (CIBEREM), Instituto de Salud Carlos III (ISCIII), Madrid, Spain; 3 Endocrinology and Nutrition Department, EASO Collaborating Centre for Obesity Management, Vall d’Hebron University Hospital, Vall d’Hebron Institut de Recerca (VHIR), Autonomous University of Barcelona, Barcelona, Spain; 4 Endocrine, Bariatric and Metabolic Surgery Unit, IFSO Centre of Excellence, Vall d’Hebron University Hospital, Vall d’Hebron Institut de Recerca (VHIR), Autonomous University of Barcelona, Barcelona, Spain; 5 Gastroenterology Department. Arnau de Vilanova University Hospital, Institut de Recerca Biomèdica (IRB) and University of Lleida, Lleida, Spain; 6 Department of Pathology and Molecular Genetics, Arnau de Vilanova University Hospital, Institut de Recerca Biomèdica (IRB) and University of Lleida, Lleida, Spain; 7 Pathology Department, Vall d’Hebron University Hospital, Vall d’Hebron Institut de Recerca (VHIR), Autonomous University of Barcelona, Barcelona, Spain; 8 Gastrointestinal Surgery Department. Arnau de Vilanova University Hospital, Institut de Recerca Biomèdica (IRB) and University of Lleida, Lleida, Spain; Università degli Studi di Palermo, ITALY

## Abstract

**Background:**

Helicobacter pylory (HP) infection has been associated to an increased rate of type 2 diabetes (T2D) and liver disease through its effect on insulin resistance and systemic inflammation. However, results are inconstant and no studies exist in morbidly obese patients, in which both insulin resistance and inflammation coexist.

**Material and Methods:**

Cross-sectional study to evaluate the relationship between HP infection and alterations in carbohydrate metabolism, lipid profile, inflammation markers, and liver disease in patients awaiting for bariatric surgery. HP infection was histologically assessed in gastric antrum biopsy from 416 subjects. Liver biopsy was also available in 93 subjects.

**Results:**

Both impaired fasting glucose and T2D were similar when comparing subjects with and without HP infection (24.2% vs. 22%, p = 0.290 and 29.4% vs. 29.1%, p = 0.916, respectively), with no differences between groups in the HOMA-IR, lipid profile neither inflammatory parameters. However, HP infection was higher among subjects with a BMI ≥ 40.0 kg/m^2^ in comparison with lower degrees of obesity (71.7% vs. 60.0%, p = 0.041). In addition, subjects without HP infection showed higher degrees of steatosis (44.1±26.4% vs. 32.0±20.7%, p = 0.038), as well as a lower prevalence of non-alcoholic steatohepatitis (9.3% vs. 30.7%, p = 0.023).

**Conclusions:**

In patients with morbid obesity, HP infection does not seem to be associated with abnormal carbohydrate metabolism. In addition, less advanced degrees of non-alcoholic fatty disease were observed. We suggest that low-grade inflammation that accompanies obesity mitigates the diabetogenic effect of HP, so the presence of obesity should be considered in studies that evaluate the HP metabolic effects.

## Introduction

Helicobacter pylori (HP) is a micro-aerofilic, flagellated, Gram-negative pathogen, highly adapted for survival in the acidic pH [1]. HP successfully colonizes human stomach, and is present in more than half of worldwide population, being more common in societies with low socioeconomic development [1]. Colonization of the gastric mucosa by HP is acquired early in life, it will persist unless antibiotic therapy, and induce not only gastric diseases, but also metabolic manifestations [2, 3]. In this way, HP seropositivity has been related with an increased rate of incident type 2 diabetes (T2D) after 10 years’ follow-up period [4]. In accordance with this finding, cross-sectional studies have also found a positive association between HP infection and the prevalence of T2D in selected populations [5, 6]. However, in contrast with these results, other studies have failed to report this relationship [7, 8]. Considering that HP does not pass into systemic circulation, it has been suggested that extragastric manifestations are mediated by insulin resistance, in relation with proinflammatory cytokines and acute phase reactants produced by the inflamed mucosa [3].

HP infection has also been associated with food intake regulation through the effect on hormones that regulate appetite, with lower production of ghrelin, and higher concentrations of leptin in comparison with HP negative subjects [9, 10]. In this way, existing data show that HP eradication in patients with peptic ulcer disease significantly increases the incidence of obesity [10, 11]. In addition, morbidly obese subjects from United States showed a 1.7-fold increase in HP infection in comparison with non-obese controls (95% CI: 1.3 to 2.2) [12].

Finally, a role for HP infection in the development of non-alcoholic fatty liver disease (NAFLD) has also been suggested [13]. In fact, the inoculation with HP in an animal model induced a significant increase in the fibrotic score and aminotransferase activity [14]. However, studies with human liver biopsy according to HP infection are scare.

On this basis, our aim was to go deeper on assessing the relationship between HP infection and alterations in carbohydrate metabolism, lipid profile and inflammatory markers in a homogeneous sample of 416 subjects with morbid obesity. For this purpose, we have chosen this population of severely obese subjects in which metabolic disruptions and low-grade chronic inflammation are usual features. In addition, the relationship between HP and NAFLD was assessed in 93 subjects with both gastric and liver biopsies.

## Material and Methods

### Ethics statement

Informed written consent was obtained from all participants, and the human ethics committee of the two participating University Hospitals (Anau de Vilanova and Vall d’Hebron) approved the study. An additional informed written consent was obtained from patients who underwent liver biopsy.

### Description of patients

A total of 416 patients of Caucasian origin attending to the outpatient Obesity Unit of two University hospitals (Arnau de Vilanova and Vall d’Hebron) were enrolled at the time of a regular visit between July 2010 and June 2014. Patients were recruited irrespective of the presence of gastrointestinal symptoms. The study was conducted according to the ethical guidelines of the Helsinki Declaration.

The next exclusion criteria were considered: type 1 diabetes, previous HP eradication therapy, requirements of corticosteroid treatment, malignancy, end stage renal disease, chronic liver disease, consuming more than 200 g of alcohol per week, or absence of pathology data for the study.

All patients underwent an established protocol before surgery, including upper gastrointestinal endoscopy and gastric antrum biopsy. HP status was evaluated in each patient by histopathology examination by the Diff-Quick method [15]. Laboratory tests included fasting plasma glucose, glycated hemoglobin A1c (HbA1c), fasting insulin, liver function tests (alanine aminotransferase, aspartate aminotransferase, and gamma-glutamyl transpeptidase), lipid profile (triglycerides, high and low-density lipoprotein), and markers of inflammation [(leukocyte count, ferritin, erythrocyte sedimentation rate (ESR), and C-reactive protein (CRP)]. Demographic data (gender and age), BMI and waist circumference, and prior diagnosis of T2D were also collected. Insulin resistance was estimated using the homeostatic model assessment method (HOMA) [16]. T2D and impaired fasting glucose (IFG) were defined according to the criteria recommended by the Expert Committee on the Diagnosis and Classification of Diabetes [17].

Liver biopsy was taken at the time of bariatric surgery with a Hepafix needle in 93 subjects. All biopsies were at least 2 cm long and contained at least eight portal tracts. Liver specimens were stained with hematoxylin eosin, picrosiriums for fibrosis and periodic acid Schiff (PAS) with diastase to help clarify the degree of inflammation. Liver histology was assessed using a systemic approach of necroinflammatory grading and fibrosis staging as described by Brunt et al [18] and modified by Kleine et al [19]. Individual histological features were observed and scored separately. Finally, all were graded and staged for non-alcoholic steatohepatitis (NASH) according to the system proposed at the American Association for the Study of Liver Diseases single topic conference in September 2002 [20].

### Statistical analysis

The normal distribution of the variables was evaluated using the Kolmogorov-Smirnov test. Data were expressed either as the mean ± SD, percentage or median (total range). Comparisons between groups were performed using the Student’s t test or the Mann-Whitney U test for continuous variables, as well as the χ^2^ test for categorical variables. All “p” values were based on a two-sided test of statistical significance. Significance was accepted at the level of p<0.05. Statistical analyses were performed using the SSPS statistical package (SPSS, Chicago, IL, USA).

## Results

The prevalence of HP infection in the whole study population was 69.5%, without differences between those with and without T2D (69.1% vs. 69.6%, p = 0.908). However, HP infection was significantly higher among subjects with a BMI ≥ 40.0 kg/m^2^ in comparison with lower degrees of obesity (71.7% vs. 60.0%, p = 0.041).

On the other hand, the prevalence of both IFG and T2D was similar when comparing subjects with and without HP infection (24.2% vs. 22.0%, p = 0.290 and 29.4% vs. 29.9%, p = 0.916, respectively). In addition, no differences between subjects HP + and HP- in their lipid profile, liver function test neither inflammatory parameters were found (Table 1).

**Table 1:** Main clinical characteristics, metabolic data, liver function, lipid profile, and inflammatory parameters according to HP positivity.

	HP positive	HP negative	P value
**N**	289	127	-
**Women, n (%)**	210 (72.6)	94 (74.0)	0.775
**Age (yrs)**	45.9 ± 9.8	44.6 ± 11.0	0.261
**BMI (kg/m**^**2**^**)**	44.4 ± 5.1	44.1 ± 5.6	0.600
**IFG, n (%)**	70 (24.2)	28 (22.0)	0.290
**T2D, n (%)**	85 (29.4)	38 (29.9)	0.916
**Fasting plasma glucose (mmol/l)**	6.4 ± 2.5	6.1 ± 1.9	0.304
**HbA1c (%)**	5,7 (4–12.3)	5.6 (4.7–11.4)	0.498
**HOMA-IR** ^a^	5.9 ± 7.0	5.6 ± 3.5	0.691
**AST (IU)**	22.5 ± 8.5	27.4 ± 16.7	0.316
**ALT (IU)**	25.0 ± 12.7	29.0 ± 23.6	0.264
**GGT (IU)**	36.8 ± 40.7	44.1 ± 31.2	0.375
**Total cholesterol (mmol/l)**	5.9 ± 1.0	5.0 ± 0.9	0.128
**HDL cholesterol (mmol/l)**	1.2 ± 0.2	1.1 ± 0.2	0.121
**LDL cholesterol (mmol/l)**	3.1 ± 1.9	3.1 ± 0.8	0.409
**Triglycerides (mmol/l)**	1.7 ± 0.9	1.7 ± 0.9	0.623
**C-reactive protein (mg/l)**	1.0 ± 0.9	0.8 ± 0.6	0.130
**Erythrocyte sedimentation rate (mm/h)**	27.6 ± 19.8	24.5 ± 16.0	0.192
**White blood cell count (x10**^**9**^**/l)**	8.2 ± 2.2	8.0 ± 2.0	0.531
**Ferritin (ng/ml)**	190.1 (8–591)	186.2 (8–550)	0.755

BMI: body mass index; IFG: impaired fasting glucose; T2D: type 2 diabetes; AST: aspartate aminotransferase; ALT: alanine aminotransferase; GGT: gamma-glutamyl transpeptidase.

^a^: HOMA-IR was calculated only in subjects without T2D.

When the subgroup of non-diabetic patients was evaluated (n = 293), HP infection was not associated with higher levels of insulin resistance (HOMA-IR: 5.6 ± 7.6 vs. 5.6 ± 3.4, p = 0.993) neither fasting plasma glucose (98.1 ± 14.6 vs. 95.0 ± 10.6 mg/dl, p = 0.079) or HbA1c (6.1 ± 6.7% vs. 5.8 ± 5.4%, p = 0.733).

**Fig 1:**
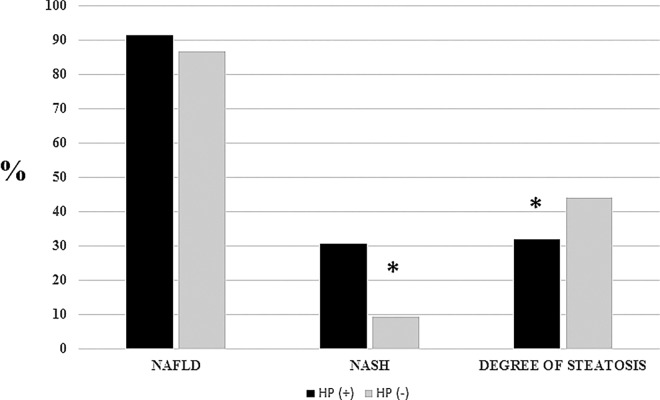
Prevalence of non-alcoholic fatty liver disease, non-alcoholic steatohepatitis, and the degree of steatosis according to the presence of Hellicobacter Pylori infection in the subgroup of 93 patients with both gastric and liver biopsies. HP: Helicobacter Pylori; NAFLD: non-alcoholic fatty liver disease; NASH: non-alcoholic steatohepatitis.

Finally, the prevalence of NAFLD was similar between subjects with and without HP infection (91.4% vs 86.6%, p = 0.499). However, among those subjects with NAFLD, those without HP infection presented a lower percentage of NASH (9.3% vs. 30.7%, p = 0.023) as well as higher degree of steatosis (44.1 ± 26.4% vs. 32.0 ± 20.7, p = 0.038) (Fig 1).

## Discussion

We failed to observe any difference in the prevalence of carbohydrate abnormalities according to the presence of histologically proven HP infection in a subgroup of subjects with morbid obesity. In recent years HP infection has been associated with increased insulin resistance [21, 22], as well as with a greater incidence and prevalence of T2D and metabolic syndrome [4, 23, 24]. However, this link appears to be inconstant, mainly attributed to heterogeneous study populations, with or without gastrointestinal symptoms, and the diversity of the diagnostic techniques (histology vs. serology) to confirm HP infection [25].

Regarding insulin resistance, a recent systematic review confirmed the positive epidemiological association with HP infection [21]. Similarly, among non-diabetic subjects, those with HOMA-IR ≥ 2.5 showed a significantly higher rate of HP seropositivity compared with non-insulin resistant subjects [22]. These data contrast with our results in morbidly obese subjects, in whom high degrees of insulin resistance were observed independently of HP status. Discordant results also emerge when the effect on HOMA-IR is evaluated after HP eradication [3, 26, 27, 28].

On the other hand, when data from participants included in the NHANES III and NHANES 1999–2000 was evaluated, there were no association between HP seropositivity and history of self-reported diabetes. Only after excluding individuals with history of diabetes and controlling for potential confounder factors, HP seropositivity was positively associated with higher mean HbA1c levels [29]. In addition, a recent meta-analysis that included more than 14,000 patients from forty-one observational studies communicated an OR for HP infection of 1.33 (95% CI: 1.08–1.64; p = 0.008) among patients with diabetes [30]. Nevertheless, our results do not confirm these data, as no differences in HbA1c were detected among morbidly obese patients without DM according to HP infection, as well as no differences appeared in the prevalence of T2D when the whole population was evaluated.

The evaluation by indirect methods like anti-HP titer has the disadvantage that the serological positivity can persist despite the bacterial eradication, resulting in subject misclassification [31]. Therefore, in a recent study of 5,889 subjects, the association between metabolic syndrome and HP infection seemed to be higher with histologic positivity for HP [adjusted odds ratio = 1.26; 95% CI 1.08–1.48) than serologic positivity (adjusted odds ratio = 1.12; 95% CI 0.95–1.32), after adjusting for age, sex, smoking status, alcohol consumption, and economic status [23]. In our study histologic positivity for HP was mandatory, reinforcing the lack of relationship between HP infection and abnormal carbohydrate metabolism in obese patients awaiting for bariatric surgery.

The prevalence of HP infection in patients with morbid obesity is highly variable, ranging between 11 and 85%. However, a higher prevalence of HP infection among bariatric patients compared to the general population [12, 32] and similar non-obese groups [33–35] has been reported. For example, the prevalence rate of 48% HP seropositivity in the general population from United States increased significantly to 61% in morbidly obese patients [12]. Also in a study conducted with the NHANES population (13,489 participants) it was observed that the effect of HP on impaired glucose tolerance could be enhanced by a high BMI [29]. Similarly, the rate of HP infection significantly increased from 60.0% in patients with a BMI < 40 kg/m2 to 71.7% when the BMI was equal or higher than 40.0 kg/m^2^.

The contributing effect of HP infection to the development of NAFLD has also been suggested, as well as for the progression from hepatic steatosis to NASH [13, 36–38]. In addition to insulin resistance, invasion of HP in the small bowel mucosa might increase intestinal permeability and facilitate the passage of bacterial endotoxins via the portal vein to the liver [13]. In this way, the severity of steatosis in humans has been correlated with the disruption of intercellular tight junctions in the gut, increasing intestinal permeability, and the intestinal bacterial overgrowth in the small bowel microbiota [39]. In a recent work, the prevalence of NASH, the total NAFLD activity score, and the grade of hepatocyte ballooning were significantly higher in patients positive for anti-HP immunoglobulin G than in those negative [36]. Similarly, higher rates of anti-HP IgG were detected in patients with biopsy-proven NAFLD compared to control group [38]. Despite this, a similar prevalence of NAFLD among patients with and without HP infection was detected in our population of morbidly obese subjects. In addition, an unexpected finding was observed in those individuals with NAFLD: obese patients with histologic positivity for HP showed higher degrees of liver steatosis and lower prevalence of NASH than subjects without HP infection. Our results are in concordance with those observed in a large-scale cross-sectional study performed with Japanese adults in whom body mass index, platelet count, and serum ALT, but not HP infection, were positively associated with NAFLD diagnosed based on ultrasound methodology [40]. As imaging has limited diagnostic value for NAFLD, the prediction of fatty liver has been investigated through various surrogate scores, including the fatty liver index (FLI), and the hepatic steatosis index (HSI) in general population [41, 42]. However, those scores fail to be a valid diagnostic tool for non-alcoholic fatty liver disease (NAFLD) in our population of morbidly obese subjects [43].

The mechanism implicated in the metabolic effects related with HP infection seems to be independent of the clinical manifestations and related with a systemic low-grade inflammatory process initiated at the gastric level [1, 44]. In this way, HP has been correlated with an increased release of pro-inflammatory cytokines and acute phase proteins, such as CRP, IL-6, and TNF-α [45–47] which have not been confirmed in our study with morbidly obese subjects. Similarly, in a 10-years prospective cohort study, among 782 diabetes-free elderly Latino subjects, baseline serological HP infection, but no inflammatory cytokines (i.e., CRP and IL-6), was associated with a 2.7-fold increase in risk to develop diabetes compared with individuals without the infection [4].

Some limitations have to be considered when analyzing our results. First, we have not considered the presence of gastrointestinal symptoms in HP infected patients, what has been considered as a confounding factor. Second, our study has evaluated not only very obese but also young subjects. Despite the possibility that a longer period with an active HP infection could enhance its systemic effects cannot be discharged, we believe this is not a determinant factor in our results since HP infection is acquired early in life.

## Conclusions

In conclusion, in view of the results of our study, we can suggest that data observed in normoweight and overweight population does not seems to be true in patients with morbidly obesity, in whom HP infection does not seem to be associated with a higher prevalence of abnormal carbohydrate metabolism. It is possible that low-grade inflammation that accompanies obesity mitigates the diabetogenic effect of HP in this population, so the presence of obese patients should be considered in studies that seek to evaluate the HP metabolic effect.
